# Supplementary figures and images for: The NF-κB Factor Relish maintains blood progenitor homeostasis in the developing Drosophila lymph gland
Source: PLoS Genet. 2024 Sep 9;20(9):e1011403. doi: 10.1371/journal.pgen.1011403 (PMC11424005; doi:10.1371/journal.pgen.1011403)

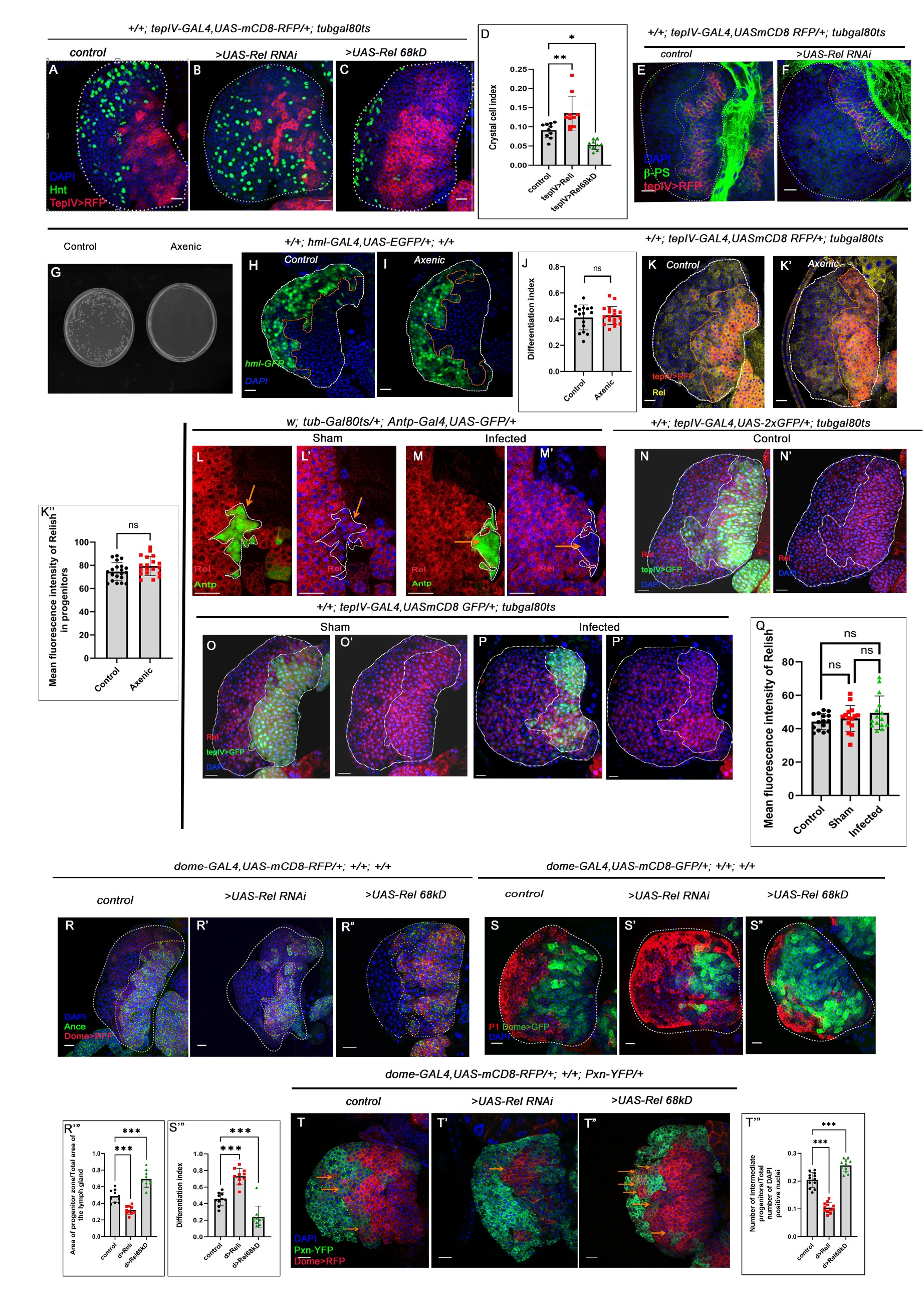

Supplement: S1 Fig — (A-C) Progenitor-specific loss of Rel increased crystal cell index (Hnt, green) (B) whereas overexpression of Rel resulted in a decrease (C) compared to control (A). (D) Differentiation index (crystal cells) for genotypes (A-C) (lymph glands n = 10 for each genotype, P-values from left to right = 0.005, and = 0.016 respectively). (E-F) No lamellocyte induction was observed in Rel loss from the progenitor (β-PS, green). (G) To check the presence of commensal gut microbiota, larval homogenates were spread on LB Agar plates. Compared to control where bacterial colonies were visible post incubation, axenic condition had no growth. (H-I) Differentiation status (hml>GFP) in the lymph gland from larvae reared in the axenic condition is comparable to control. (J) Differentiation index for each condition of H-I (lymph gland n≥16, P-value = 0.608). (K-K’) Progenitor-specific Relish expression remains unaltered in axenic condition (compare K with K’). (K”) The mean fluorescence intensity of Relish in progenitors for each condition of K-K’ (lymph gland n≥19, P-value = 0.066). (L-M’) Compare to sham (L–L’), a significant reduction in Relish expression (arrow) was observed in the hematopoietic niche 4 hr post-infection (M-M’). (N–P’) Compare to uninfected conditions (N–N’) and sham (O–O’), no significant change in Relish expression was observed in the hematopoietic progenitors 4 hr post-infection (P-P’). (Q) Statistical analysis of the data in (N–P’) (lymph glands n = 15 for each treatment, P-values from left to right = 0.780, = 0.173 and = 0.487, respectively). (R-R”) Another progenitor-specific driver, dome-Gal4 gave similar results like tepIV-Gal4. (R”’) The ratio of the area of progenitors to that of the total area of the primary lobe for the genotypes R-R” (lymph glands n = 10, P-values from left to right <0.001 and <0.001, respectively). (S-S”) A significant increase in terminally differentiated (P1, red) was observed in Rel loss employing dome-Gal4, whereas overexpression [file pgen.1011403.s001.tif]

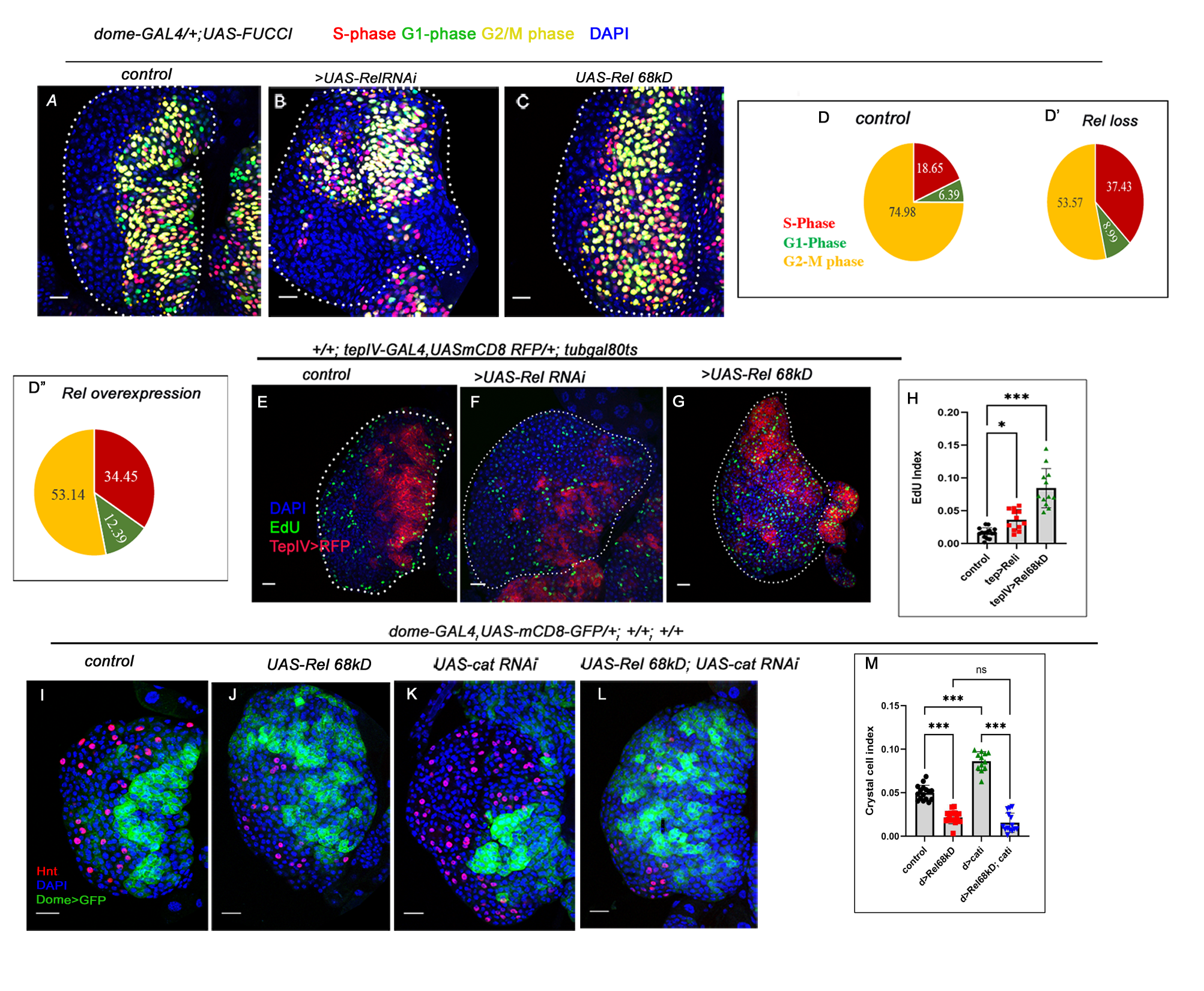

Supplement: S2 Fig — (A-C) Both loss of Rel (B) and overexpression of Rel (C) resulted in an increase of S phase (red) and G1 phase (green) at the expense of G2-M (yellow) arrested cells otherwise found in control lymph gland progenitors (A). (D-D”) Infographic representation of results in A-C. (E-G) EdU incorporation assay endorsed the findings of A-C. (H) Rate of EdU incorporation in genotypes E-G (lymph glands n≥12, P-values from left to right <0.03 for control versus UAS-Reli and <0.001 for control versus UAS-Rel68kD). (I-L) Compared to control (I), UAS-cat RNAi expression in the progenitors increases crystal cell differentiation (K). The co-expression of UAS-cat RNAi and UAS-Rel 68kD (L) is unable to rescue the halt in differentiation (crystal cells), which is observed in Rel overexpression (J). (M) Differentiation index (crystal cells) for genotypes I-L (lymph glands n≥12, P-values from left to right <0.001, <0.001, = 0.298 and <0.001). Genotypes are as mentioned. Each dot in the graph represents individual values, Data expressed as mean ±SD. Statistical analysis: Tukey’s multiple comparison tests. P-Value of <0.05, <0.01 and<0.001, mentioned as *, **, *** respectively. Scale bar: 20μ, DAPI: nucleus. (TIF) [file pgen.1011403.s002.tif]

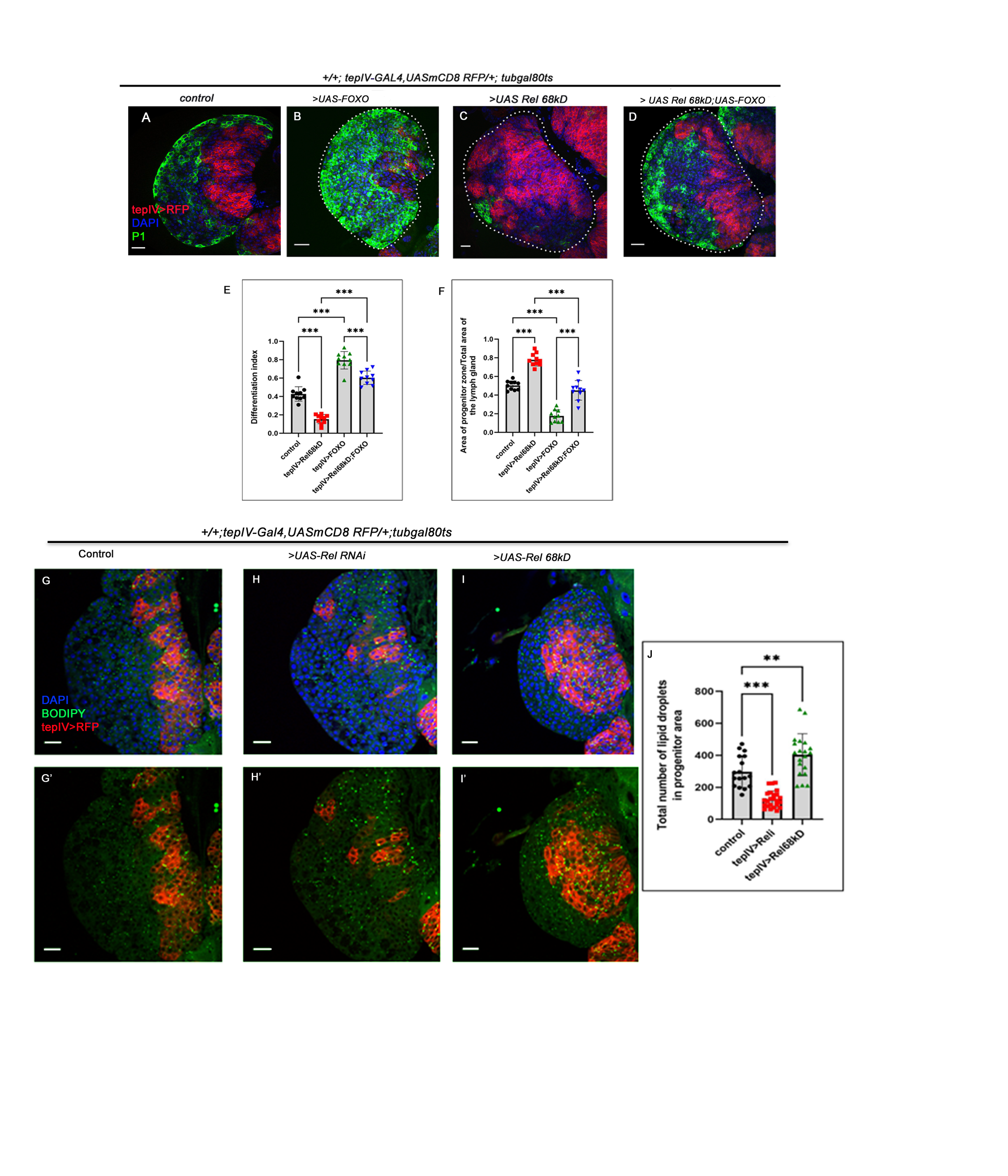

Supplement: S3 Fig — (A-D) Compared to control (A), co-expression of FOXO and Rel 68 kD rescues the halt in differentiation (D), which was otherwise observed in Rel overexpression scenario (C). Upregulating FOXO activity alone resulted in ectopic differentiation (B). (E) Differentiation index for genotypes A-D (lymph glands n = 10, P-values from left to right <0.001, <0.001, <0.001 and <0.001, for UAS-Rel68kD versus UAS-Rel 68kD; UAS-FOXO). (F) The ratio of the area of progenitors to that of the total area of the primary lobe for the genotypes A-D (lymph glands n = 10, P-values from left to right <0.001, <0.001, <0.001 and <0.001, respectively). (G-I’) Neutral lipid content visualized by BODIPY in the progenitors, compared to control lymph glands (G-G’) a drastic reduction in lipid accumulation was observed in the Rel loss condition (H-H’), whereas Rel overexpression increased the number of lipid droplets (I-I’). (J) Total number of lipid droplets in the progenitors G-I’ (lymph glands n≥17, P-values. from left to right <0.001 and <0 .005 respectively). Genotypes are as mentioned. Each dot in the graph represents individual values, Data expressed as mean ± SD. Statistical analysis: Tukey’s multiple comparison tests. P- Value of <0.05,<0.01 and<0.001, mentioned as *, **, *** respectively. Scale bar: 20μ, DAPI: nucleus. (TIF) [file pgen.1011403.s003.tif]

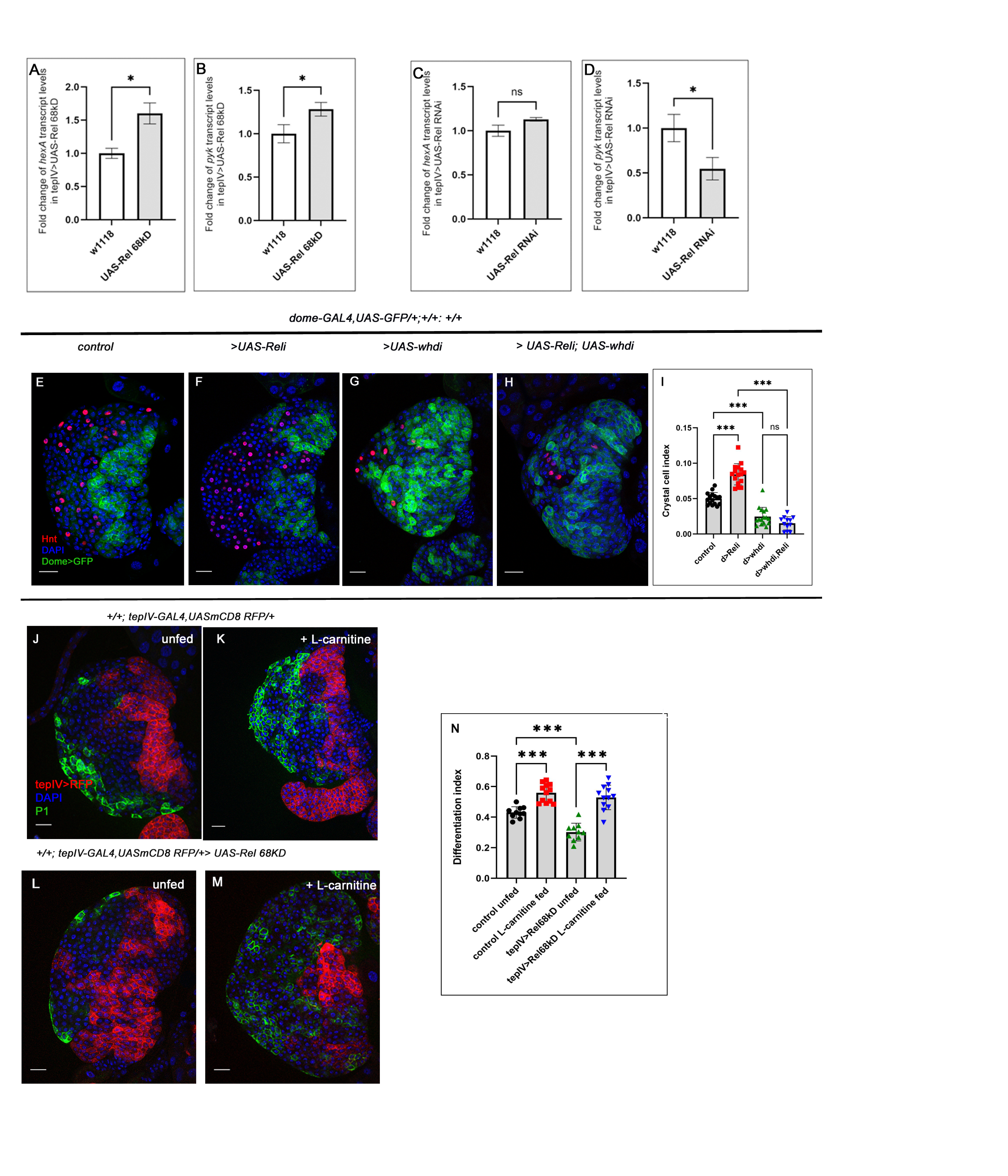

Supplement: S4 Fig — (A-D) Rel overexpression causes increments of hexA and pyk transcript levels respectively (A and B) (N = 3, P-values from left to right = 0.011 and = 0.023, respectively. The level of hexA remains unchanged (C) upon progenitor-specific Rel loss, whereas pyk transcripts decreases (D) (N = 3, P-values from left to right = 0.0593 and = 0.0175, respectively). The RNA was obtained from FACS-sorted progenitors via GFP expression using tepIV-GAL4>UAS-2XEGFP. (E-H) Compared to control (E), downregulating FAO using UAS-whd RNAi in Rel loss genetic background rescues the excessive differentiation: crystal cells (H), which was otherwise observed in Rel loss from progenitors (F). Downregulating whd alone results in a decline in crystal cell number (G). (I) Differentiation index (crystal cells) for genotypes, (E-H) (lymph glands n≥12, P-values from left to right <0.001, <0.001, <0.001 and = 0.169 respectively). (J-M) Feeding the larvae with L-carnitine supplemented food in both control (K) and progenitor-specific Rel overexpression (M) caused precocious differentiation, which was otherwise not observed in progenitor-specific Rel overexpression (L) and unfed control (J). (N) Differentiation index for genotypes J-M (lymph glands n≥10, P-values from left to right <0.001, <0.001 and <0.001, respectively). Genotypes are as mentioned. Each dot in the graph represents individual values, Data expressed as mean± SD. Statistical analysis: q-PCR: Unpaired t-test with Welch’s correction and rest Tukey’s multiple comparisons tests. P-Value of <0.05,<0.01 and<0.001, mentioned as *, **, *** respectively. Scale bar: 20μ, DAPI: nucleus. (TIF) [file pgen.1011403.s004.tif]
