# Supplementary material for: The NF-κB Factor Relish maintains blood progenitor homeostasis in the developing Drosophila lymph gland
Source: PLoS Genet. 2024 Sep 9;20(9):e1011403. doi: 10.1371/journal.pgen.1011403 (PMC11424005; doi:10.1371/journal.pgen.1011403)
Supplement: S1 Table — (DOCX) [file pgen.1011403.s005.docx]

**S1 Table: Fly Stocks used in the current study**

| **Fly Stock** | **Source** |
| --- | --- |
| *D. melanogaster. w^1118^* | Bloomington Drosophila Stock Center (#BL3605) |
| *D. melanogaster. y1 w* P{GawB}domePG14/FM7a* | Bloomington Drosophila Stock Center (#BL81010) |
| *D. melanogaster. y[*] w[*]; P{w[+mW.hs]=GawB}NP7379 / CyO, P{w[-]=UAS-*  *lacZ.UW14}UW14* | Kyoto Stock Center (DGGR#105442) |
| *D. melanogaster. CHIZ GAL4* | U. Banerjee[1] |
| *D. melanogaster. y1 sc* v1 sev21;P{TRiP.HMS00070}attP2* | Bloomington Drosophila Stock Center (#BL33661) |
| *D. melanogaster. w*; P{UAS-FLAG-Rel.68}i21-B; TM2/TM6C,Sb1* | Bloomington Drosophila Stock Center (#BL55778) |
| *D. melanogaster. y1 v1; M{WKO.P1-E6}ZH-86Fb* | Bloomington Drosophila Stock Center (#BL84191) |
| *D. melanogaster. w[1118]; PBac{802.P.SVS- 2}Pxn[CPTI003897]* | KYOTO Stock Center (DGGR#115452) |
| *D. melanogaster. w1118; P{UAS-GFP.E2f1.1-230}32 P{UAS-mRFP1.NLS.CycB.1-266}19/CyO, P{en1}wgen11; MKRS/TM6B, Tb1* | Bloomington Drosophila Stock Center (#BL55121) |
| *D. melanogaster: p{gstD-GFP} (II)* | Prof. D Bohmann [2] |
| *D. melanogaster. y1 w*; P{UAS-mCD8.mRFP.LG}18a* | Bloomington Drosophila Stock Center (#BL27398) |
| *D. melanogaster. y1 w*; P{UAS-mCD8::GFP.L}LL5, P{UAS- mCD8::GFP.L}2* | Bloomington Drosophila Stock Center (#BL5137) |
| *D. melanogaster. w*; P{UAS- 2xEGFP}AH2* | Bloomington Drosophila Stock Center (#BL6874) |
| *D. melanogaster. y1 v1; P{TRiP.JF02173}attP2* | Bloomington Drosophila Stock Center (#BL31894) |
| *D. melanogaster. w[1118]; PBac{566.P.SVS-1}CG3902[CPTI100004]* | KYOTO Stock Center ( DGGR#115356) |
| *D. melanogaster. y1 w*; P{UAS- foxo.P}2* | Bloomington Drosophila Stock Center (#BL9575) |
| *D. melanogaster. y1 v1; P{ TRiP.HMJ02053}attP40* | Bloomington Drosophila Stock Center (#BL53377) |
| *D. melanogaster. w1118 P{UAS- bsk.DN}2* | Bloomington Drosophila Stock Center (#BL6409) |
| *D. melanogaster. whd[1]* | Bloomington Drosophila Stock Center (#BL441) |
| *D. melanogaster. y1 v1; P{TRiP.HMS00040}attP2/TM3, Sb1* | Bloomington Drosophila Stock Center (#BL34066) |
| *D. melanogaster. hsflp; act>CD2>Gal4, UAS GFP* | Pradip Sinha [3] |
| *D. melanogaster. pCol85-Gal4* | M. Crozatier [4] |

1. Spratford CM, Goins LM, Chi F, Girard JR, Macias SN, Ho VW, et al. Intermediate progenitor cells provide a transition between hematopoietic progenitors and their differentiated descendants. Development. 2021;148(24). Epub 20211217. doi: 10.1242/dev.200216. PubMed PMID: 34918741; PubMed Central PMCID: PMCPMC8722385.

2. Sykiotis GP, Bohmann D. Keap1/Nrf2 signaling regulates oxidative stress tolerance and lifespan in Drosophila. Dev Cell. 2008;14(1):76-85. doi: 10.1016/j.devcel.2007.12.002. PubMed PMID: 18194654; PubMed Central PMCID: PMCPMC2257869.

3. Bhattacharya R, Kumari J, Banerjee S, Tripathi J, Mohan N, Sinha P. Hippo effector, Yorkie, is a Tumor Suppressor in Select Drosophila Squamous Epithelia2023.

4. Letourneau M, Lapraz F, Sharma A, Vanzo N, Waltzer L, Crozatier M. Drosophila hematopoiesis under normal conditions and in response to immune stress. FEBS Lett. 2016;590(22):4034-51. Epub 20160806. doi: 10.1002/1873-3468.12327. PubMed PMID: 27455465.
