# Supplementary material for: The NF-κB Factor Relish maintains blood progenitor homeostasis in the developing Drosophila lymph gland
Source: PLoS Genet. 2024 Sep 9;20(9):e1011403. doi: 10.1371/journal.pgen.1011403 (PMC11424005; doi:10.1371/journal.pgen.1011403)
Supplement: S2 Table — (DOCX) [file pgen.1011403.s006.docx]

**S2 Table: List of primers used in the current study for RT-qPCR**

| **REAGENT OR RESOURCE** | **DESIGNATION** | **SOURCE** | **IDENTIFIER** | **ADDITIONAL INFORMATION** |
| --- | --- | --- | --- | --- |
| Sequence based reagent | rp49_F | This Paper | PCR primers | GCCCACCGGATTCAAGAAGT |
| Sequence based reagent | rp49_R | This Paper | PCR primers | TTGCGCTTCTTGGAGGAGAC |
| Sequence based reagent | whd_F | Tiwari et. al., 2020 | PCR primers | GGCCAATGTGATTTCCCTGC |
| Sequence based reagent | whd_R | Tiwari et. al., 2020 | PCR primers | TGCCCTGAACCATGATAGGC |
| Sequence based reagent | hexA_F | Tiwari et. al., 2020 | PCR primers | CTGCTTCTAACGGACGAACAG |
| Sequence based reagent | hexA_R | Tiwari et. al., 2020 | PCR primers | GCCTTGGGATGTGTATCCTTGG |
| Sequence based reagent | pyk_F | This Paper | PCR primers | GTTGTTGTGCGTGGAGTTGC |
| Sequence based reagent | pyk_R | This Paper | PCR primers | GGGTACTTCGTTGGGCTTCA |
| Sequence based reagent | puc_F | This Paper | PCR primers | AGCGATACGCCACATCAGAAC |
| Sequence based reagent | puc_R | This Paper | PCR primers | ACTTGTACCGCATGACGTAGG |
