# Supplementary material for: The NF-κB Factor Relish maintains blood progenitor homeostasis in the developing Drosophila lymph gland
Source: PLoS Genet. 2024 Sep 9;20(9):e1011403. doi: 10.1371/journal.pgen.1011403 (PMC11424005; doi:10.1371/journal.pgen.1011403)
Supplement: Attachment — Submitted filename: Plos Genet review of Ramesh-Mandal.pdf [file pgen.1011403.s008.pdf]

Feb 2024 Review of
The NF-κB Factor Relish maintains blood progenitor homeostasis in the developing Drosophila lymph gland
by Ramesh et al
Submitted to PLoS Genet.

In this study, Ramesh and colleagues analyse the mechanisms that restrain blood cell progenitor differentiation in the Drosophila lymph gland. In particular, recognizing that ROS (reactive oxygen species) are both known to promote blood cell differentiation in the lymph gland and are yet widespread in this environment, the authors seek to identify factors/mechanisms that inhibit ROS-induced differentiation, restraining it to the margins of this organ. They propose the Relish NF-kB transcription factor as the key factor restraining hematopoietic differentiation: it is expressed in cells whose differentiation is blocked, and is downregulated as differentiation gets underway. A suite of loss-of-function and gain-of-function approaches supports their model.

This study builds on findings from ≥ 4 prior studies:

Park '04 showed that Relish curtails JNK pathway signalling through transcription of an unknown factor that targets Tak1 for proteasomal degradation (in Drosophila blood cell culture)

Owusu-Ansah '09 showed that ROS primes Drosophila lymph gland progenitors for differentiation.

Tiwari '20 (Mandal group) showed that fatty acid oxidation (FAO) in lymph gland progenitors is required for differentiation, via histone acetylation by acetyl CoA produced by mitochondrial metabolism of fatty acids

Ramesh '21 (Mandal group) showed that Relish functions in the niche cells of the Drosophila lymph gland to suppress differentiation, via JNK/Hh, and that Relish is downregulated during infection, allowing accelerated hematopoiesis

The current study is largely convincing in showing that progenitor-Relish inhibits blood cell differentiation through JNK/Tak/FAO mechanism, but I do not find that this study is sufficiently novel (beyond the studies listed above) or complete to warrant publication in PLoS Genetics in its current form. Below I detail some weaknesses that I feel should be addressed.

1. This paper builds on a previous one by the same authors (Ramesh et al '21 *ELife*), which found that Relish expression restrains hematopoietic differentiation in the lymph gland, and is downregulated upon immune challenge, unleashing "emergency hematopoiesis". However, that earlier paper focused on Relish expression in the niche cells within the hematopoietic organ, whereas the current paper focused on Relish expression in the progenitor cells themselves. This previous study is only glancingly mentioned (lines 127-28), and then hardly at all again. It seems then, that the same transcription factor works in both niche and progenitor cells to suppress progenitor differentiation, in both cases (it is proposed) working by inhibiting JNK signalling. The mechanisms downstream of Relish/JNK in each tissue that suppress progenitor differentiation appear to be <u>different</u> (blocking inhibitory Hh release from niche cells, vs activating fatty acid oxidation and histone acetylation cell-autonomously within progenitor cells).

Questions these parallel functions of Relish raise include:

- is Relish expression in the progenitor cells also suppressed upon immune challenge (as it is in niche cells), unleashing their differentiation?
- does (Relish-dependent) Hh expression <u>in progenitors</u> have a role in maintaining their undifferentiated state?
- Relish expression in the niche is ecdysone-dependent; is this also true of Relish in the progenitors?

2. The evidence provided that cell-autonomous suppression of progenitor blood cell differentiation by Relish works through JNK pathway suppression includes that

A. JNK target gene *puckered* is upregulated when Relish is knocked down, and suppressed when Relish is overexpressed (Fig 3A, B).

B. The precocious progenitor differentiation (and inhibition of proliferation) seen when Relish is knocked down is suppressed when the JNK pathway is simultaneously inhibited (bskDN).

The latter (B) epistatic analysis is convincing, but the data on puckered gene regulation by Relish (A) is confusing. It seems that it would have been straightforward to examine a JNK transcriptional reporter in situ (eg puc-GFP). Instead, however, the authors take a very convoluted approach. They separately dissociate cells WT vs RelishKD vs RelishOE pooled lymph glands, FACS-sort progenitors via GFP expression, and analyze puc expression by qPCR. The figure legend lists the genotypes, but since no progenitor GFP expression construct is included, there is yet more confusion in this reader.

3. The evidence that the mechanism through which loss of Relish promotes differentiation is via FAO-dependent acetyl-CoA-histone acetylation includes reference to their prior work (Tiwari '20) which showed that lymph gland loss of histone acetylases and/or acetyl-coA synthases blocked differentiation; that histone acetylation was reduced when FAO was blocked (including clonal analysis; and that dietary acetate supplementation (50 mM) restored histone acetylation and differentiation.

In the current paper, the authors take a more casual approach to showing that Relish loss promotes differentiation via histone acetylation. Instead of clonal analysis examining the effect of Relish expression levels on histone acetylation, the authors look at whole tissue RNAi. More problematic is the use of dietary acetate supplementation. Jugder et al (2021 *Immunity*) showed that dietary supplementation with the same 50 mM acetate induced Imd (Relish!) signalling in the intestine, and affected whole-organism metabolic physiology. Therefore I recommend great caution in interpreting lymph gland phenotypes resulting from acetate supplementation, because we know how much lymph gland biology is affected by immune-metabolic events elsewhere in the organism.

4. Drosophila possesses 3 NF-kB factors; in addition to Relish are Dif and Dorsal. Louradour et al 2017 eLife showed that Dif expression in the lymph gland niche is activated by ROS, and contributes indirectly to progenitor differentiation into lamellocytes. Moreover, Louradour et al indicate that EGFR signalling in the progenitors synergizes with the ROS signalling to promote differentiation, but do not clarify whether this works through JNK. The authors of the manuscript under consideration do not cite this paper. There now have been enough papers on NFkB factors in the lymph gland that each contribute some insight that there is a now need for a broader understanding of how ROS, NFkB factors, JNK interact in the lymph gland to regulate progenitor differentiation, not only into plasmatocytes, but also lamellocytes and crystal cells, both under steady-state and immune challenge circumstances. The submitted manuscript, in its current form, has too narrow of a focus to warrant acceptance into PLoS Genetics.
