# Supplementary material for: The NF-κB Factor Relish maintains blood progenitor homeostasis in the developing Drosophila lymph gland
Source: PLoS Genet. 2024 Sep 9;20(9):e1011403. doi: 10.1371/journal.pgen.1011403 (PMC11424005; doi:10.1371/journal.pgen.1011403)
Supplement: Attachment — Submitted filename: Response to Reviewers Comments (PGENETICS-D-24-00160).pdf [file pgen.1011403.s009.pdf]

Response to Reviewers Comments: (PGENETICS-D-24-00160)

Reviewer #1:

"The NF-ϰB Factor Relish maintains blood progenitor homeostasis in the developing Drosophila lymph gland" is an elegant genetic study revealing the novel role of Drosophila NF-kB-like factor Relish (Rel) in preventing hematopoietic progenitors from undergoing ROS-induced differentiation. This study addressed a significant question- what is the mechanism that protects the progenitors from entering the differentiation program in the presence of ROS that is known to trigger their differentiation? They showed that Rel loss results in the precocious differentiation of the hematopoietic progenitors, suggesting a protective role. As Rel has a well-documented role in immune response, they have provided evidence to argue that the protective role of Rel is not related to a evoke in the immune response. Importantly, Rel overexpression led to a block in progenitor differentiation, an effect opposite to that caused by Rel loss. In terms of mechanism of action, the authors have convincingly demonstrated that Rel has an inhibitory role towards JNK activation, thereby preventing progenitor differentiation. As fatty acid oxidation (FAO) acts downstream of JNK for progenitor differentiation, they went to show that FAO as well as FAO-mediated histone acetylation also act downstream of Rel in controlling progenitor differentiation program via genetic analysis and pharmacological treatment. Overall, the experiments have been executed meticulously, with proper controls, and the data are of high quality, supported by statistical analyses. Moreover, the manuscript is well-written and a pleasure to read. The findings will be of great interest to general readers of PLOS Genetics.

Thank you for the comments on our approach and the findings. Your opinions mean a lot to us, and it gives us much satisfaction that you have found our work interesting.

Major comments:

1. Rel is a transcription factor, a well-known fact that was not mentioned in this manuscript. This needs to be improved. It is unclear how Rel negatively regulates JNK pathway. Although not essential for the publication of this manuscript, the reviewer would like to see a discussion on how Rel might be linked to JNK pathway during hematopoietic progenitor lineage development.

Thanks for your suggestion. We have done the necessary edits and inclusions.

1. **Kindly refer to line 64 and line 136 in the revised manuscript where we have mentioned Rel as a transcription factor "…….. *Drosophila* NF-κB transcription factor Relish (Rel)……"**
2. **Regarding a discussion on how Rel might be linked to JNK pathway during hematopoietic progenitor lineage development.….Please refer to lines 438-448 in the discussion section.**

2. It is interesting that there is a region-specific differentiation in the outer rim of the MZ.

Any spatial regulation of Rel (ie any gradient localization of Rel from inner to outer rim)?

We thank the Reviewer for bringing up this point. ROS is the trigger for differentiation, yet the ROS-enriched progenitors do not differentiate in one go. Indeed, the region-specific differentiation in the outer rim of the MZ is brought about by the interplay of metabolic and developmental signals. Although our genetic study demonstrates that, inspired by the Reviewer's comment, we performed an expression analysis of Rel with gstD1-GFP (reporter of ROS) and CHIZ- GFP (a marker for Intermediate progenitors, where a differentiation program has been initiated). Please refer to Figure 2A-A‴ and Figure 1S-S″, T. This aspect is also discussed in text of the revised version (Lines: 216-218 for CHIZ-GFP, and 255-259 for gstD1-GFP)

This set of expression analyses further endorses our genetic data. The ROS-enriched MZ cells that are Relish positive refrain from differentiating (Figure 6E-E″). Whereas as Relish expression declines in the peripheral region of MZ, the ROS-enriched progenitors differentiate. The low Rel region can be mapped to CHIZ-GFP expressing cells, which have started differentiating.

How is it regulated by Rel?

Our genetic data reveals that Rel inhibits JNK by restraining TAK1 expression, otherwise known to activate JNK. Interestingly, the JNK activation downstream of ROS is calibrated to prevent the exhaustion of progenitors in one shot.

Has single-cell RNA sequencing been performed on the lymph gland to support the model in Fig S5K? Again, these are not essential for this manuscript, but will be good to include a discussion to point at future directions.

Our q-PCR-based transcript analyses in the current and previous publications show the presence of Rel/ JNK/FAO components in the late third instar lymph gland. We have not performed a single-cell analysis. We appreciate that the Reviewer thought this was optional for the current manuscript.

We have also worked on figure (Current Fig.7) and made it clearer to convey our results comprehensively.

Minor comments:

1. When mentioning "Lamellocytes", indicate that they are "a class of hemocytes".

   Have taken care: Please refer to Line: 190-191.

2. In "These results indicate that the ectopic differentiation observed in Rel loss might be rescued by blocking FAO and Rel simultaneously.", remove "and Rel simultaneously".

   Thanks for pointing this. We have done the necessary editing. Please refer to lines 360-362.

Reviewer 2

In this study, Ramesh and colleagues analyse the mechanisms that restrain blood cell progenitor differentiation in the Drosophila lymph gland. In particular, recognizing that ROS (reactive oxygen species) are both known to promote blood cell differentiation in the lymph gland and are yet widespread in this environment, the authors seek to identify factors/mechanisms that inhibit ROS-induced differentiation, restraining it to the margins of this organ. They propose the Relish NF-kB transcription factor as the key factor restraining hematopoietic differentiation: it is expressed in cells whose differentiation is blocked, and is downregulated as differentiation gets underway. A suite of loss-of-function and gain-of-function approaches supports their model.

This study builds on findings from ≥ 4 prior studies:

Park '04 showed that Relish curtails JNK pathway signalling through transcription of an unknown factor that targets Tak1 for proteasomal degradation (in Drosophila blood cell culture)

Owusu-Ansah '09 showed that ROS primes Drosophila lymph gland progenitors for differentiation.
Tiwari '20 (Mandal group) showed that fatty acid oxidation (FAO) in lymph gland progenitors is required for differentiation, via histone acetylation by acetyl CoA produced by mitochondrial metabolism of fatty acids

Ramesh '21 (Mandal group) showed that Relish functions in the niche cells of the Drosophila lymph gland to suppress differentiation, via JNK/Hh, and that Relish is downregulated during infection, allowing accelerated hematopoiesis

The current study is largely convincing in showing that progenitor-Relish inhibits blood cell differentiation through JNK/Tak/FAO mechanism, but I do not find that this study is sufficiently novel (beyond the studies listed above) or complete to warrant publication in PLoS Genetics in its current form. Below I detail some weaknesses that I feel should be addressed.

Thanks for your time and finding our study convincing.

1. This paper builds on a previous one by the same authors (Ramesh et al '21 *ELife*), which found that Relish expression restrains hematopoietic differentiation in the lymph gland, and is downregulated upon immune challenge, unleashing "emergency hematopoiesis". However, that earlier paper focused on Relish expression in the niche cells within the hematopoietic organ, whereas the current paper focused on Relish expression in the

progenitor cells themselves. This previous study is only glancingly mentioned (lines 127-28), and then hardly at all again. It seems then, that the same transcription factor works in both niche and progenitor cells to suppress progenitor differentiation, in both cases (it is proposed) working by inhibiting JNK signalling. The mechanisms downstream of Relish/JNK in each tissue that suppress progenitor differentiation appear to be different (blocking inhibitory Hh release from niche cells, vs activating fatty acid oxidation and histone acetylation cell-autonomously within progenitor cells).

Thank you for clearly summarising the diverse mechanism Rel adopts to maintain progenitor (from niche vs MZ). As suggested, we have included the observations from the previous niche-related study to compare and contrast the current observations (Please refer to Lines 196-205 in the revised manuscript and Figure S1L-M′).

Questions these parallel functions of Relish raise include:

- is Relish expression in the progenitor cells also suppressed upon immune challenge (as it is in niche cells), unleashing their differentiation?
-

The newly included observations in Fig S1 clearly show that post-infection, while Rel expression declines in niche (marked by Antp>GFP), MZ-specific expression remains unaltered *(Fig S1L-M′)*.
To validate further, we repeated the experiments in the genotype wherein MZ was positively labelled. Relish expression was not affected in the lymph gland progenitors of infected larvae compared to the sham and control lymph glands *(Compare FigS1 N-N′, O-O′* and *P-P′* and *S1Q)*.
This along with our axenic data (S1G-S1K′′), further validates that the progenitor-specific expression of Relish is not susceptible to the pathophysiological state of the organism but instead seems to serve as a developmental cue.
-
- does (Relish-dependent) Hh expression in progenitors have a role in maintaining their undifferentiated state?

- **Hh produced by the niche is sensed by the progenitors. The transcriptional activator of Hh signalling, Ci^{155} can be detected in the progenitors.**

- **Following the Reviewer's suggestion, we analysed Ci^{155} expression in the progenitors upon Relish loss. Our analyses reveal that there is no decline in Ci^{155} expression, upon loss of Relish from the progenitors. This finding is in contrast to our previous observation wherein we observed that loss of Relish from the PSC impacts Ci^{155} expression in the progenitors (Ramesh et al., 2021). Thus, we can conclude that Hh from the niche is not a part of this regulatory network that Rel evokes in the progenitors.**
  Please find the images and quantitative analysis of the above data.

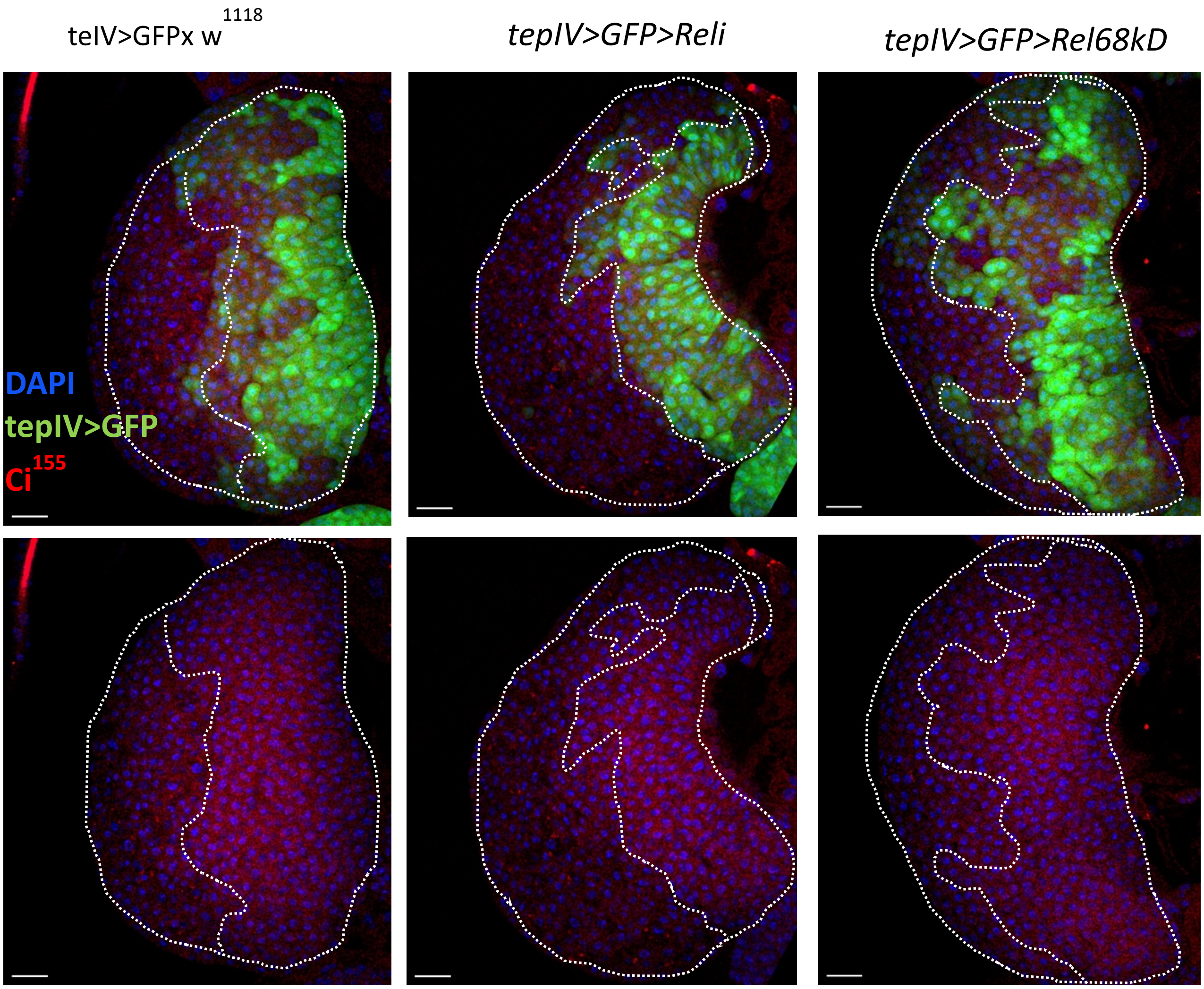
teIV>GFPx w1118
tepIV>GFP>Reli
tepIV>GFP>Rel68kD
DAPI
tepIV>GFP
Ci155

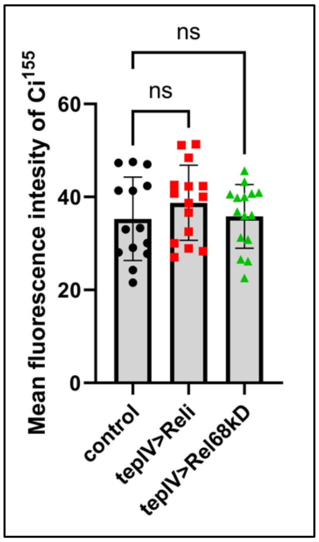
Mean fluorescence intesity of Ci155
ns
ns
60
40
20
0
control
tepIV>Reli
tepIV>Rel68kD

- Relish expression in the niche is ecdysone-dependent; is this also true of Relish in the progenitors?
-
- **To address this query, we downregulated Ecdysone signalling from the progenitors by expressing EcRDN and assayed for the expression level of Relish.**
- **As evident from the images below, although EcRDN has a phenotype in the MZ (the progenitor area is significantly small compared to control), the level of Rel expression is comparable to control.**
- **This is a stark difference to Rel expression in PSC/niche wherein Rel expression is Ecdysone dependent (Ramesh et al., 2021). Thus, we conclude that Rel expression in the progenitors is not ecdysone dependent.**
-

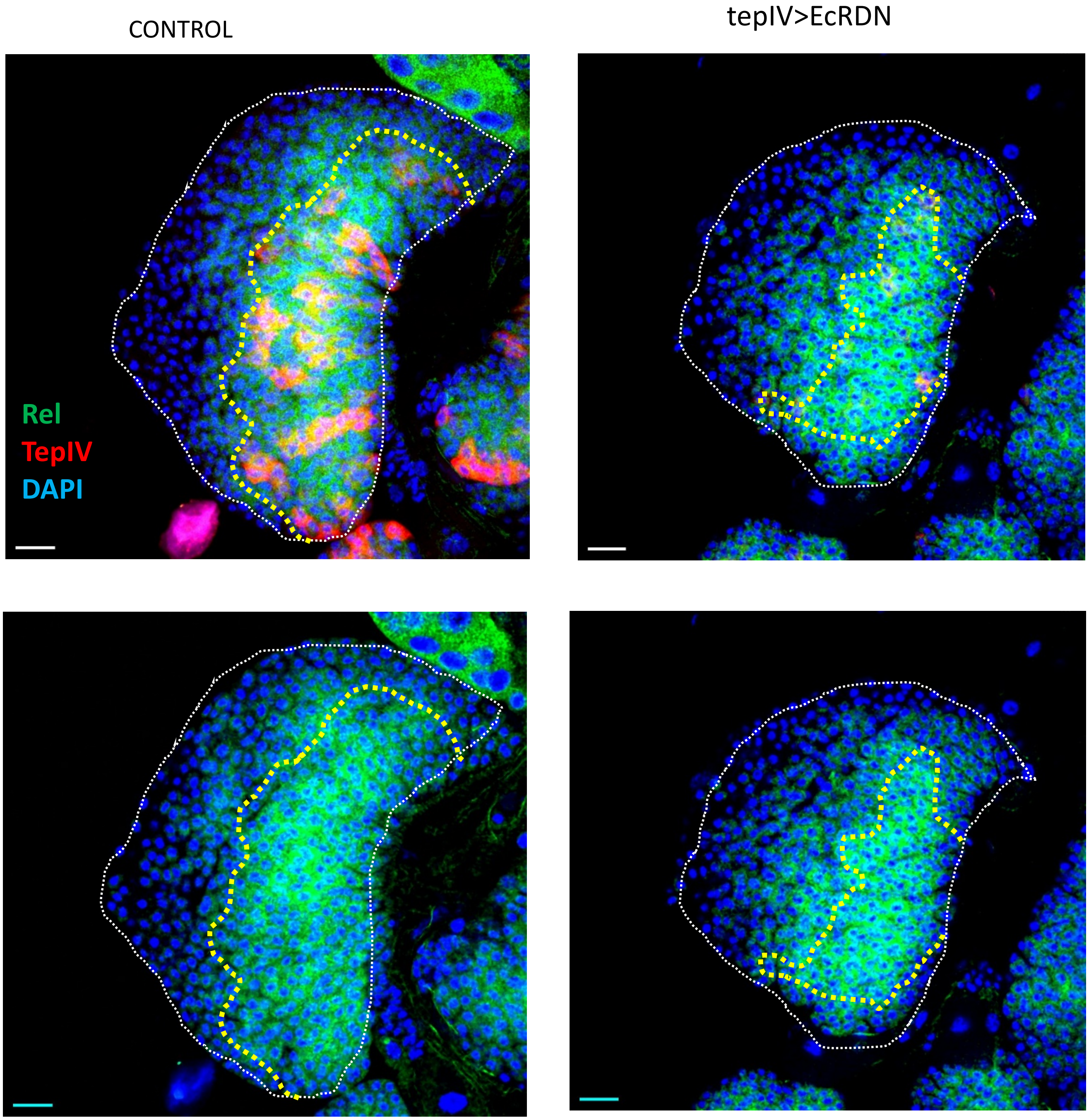

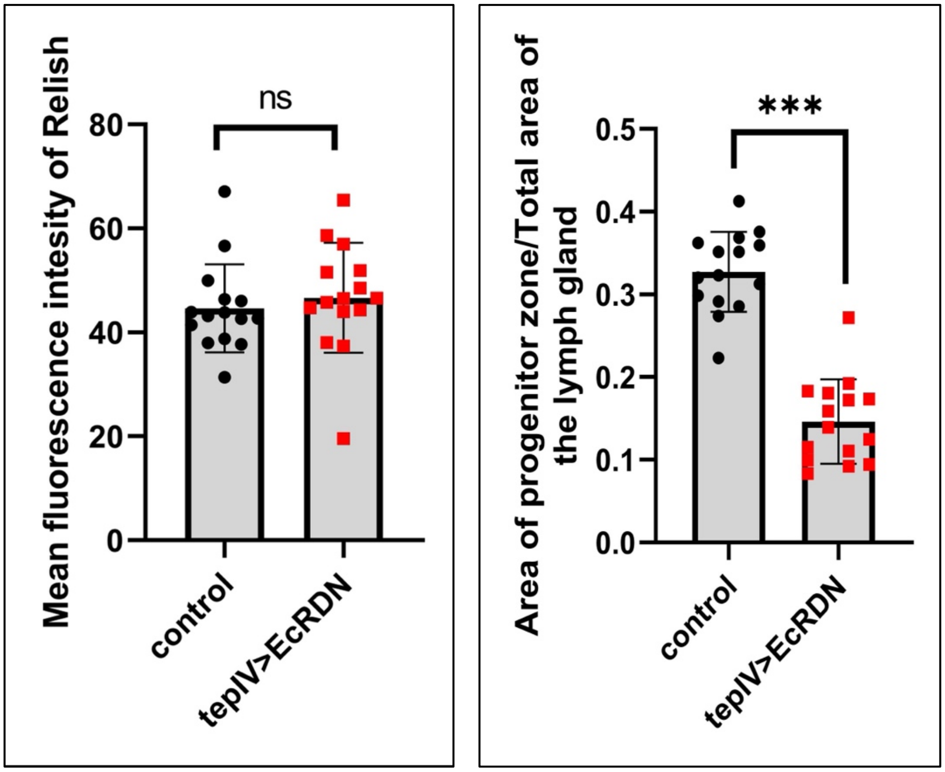

2. The evidence provided that cell-autonomous suppression of progenitor blood cell differentiation by Relish works through JNK pathway suppression includes that

A. JNK target gene *puckered* is upregulated when Relish is knocked down, and suppressed when Relish is overexpressed (Fig 3A, B).

B. The precocious progenitor differentiation (and inhibition of proliferation) seen when Relish is knocked down is suppressed when the JNK pathway is simultaneously inhibited (bskDN).

The latter (B) epistatic analysis is convincing, but the data on puckered gene regulation by Relish (A) is confusing. It seems that it would have been straightforward to examine a JNK transcriptional reporter in situ (eg puc-GFP). Instead, however, the authors take a very convoluted approach.

Thanks for your comments on epistasis analysis. However, we beg to disagree with the Reviewer's comment that transcript analysis from the pure population is a convoluted approach.

We have previously done *puc-lacZ* expression upon Rel genetic perturbation. The upregulated *puc-lacZ* expression upon Rel loss was evident in the analysis (Please see the figure below). However, instead of solely depending on the reporter assay, the transcriptional activation of the JNK pathway will be more relevant and convincing. Thus, we FACS-sort progenitors via GFP expression from WT vs Relish RNAi vs Relish^{OE} pooled lymph glands and analyzed *puc* expression by qPCR.

tepIV>RFP; +/pucLACZ

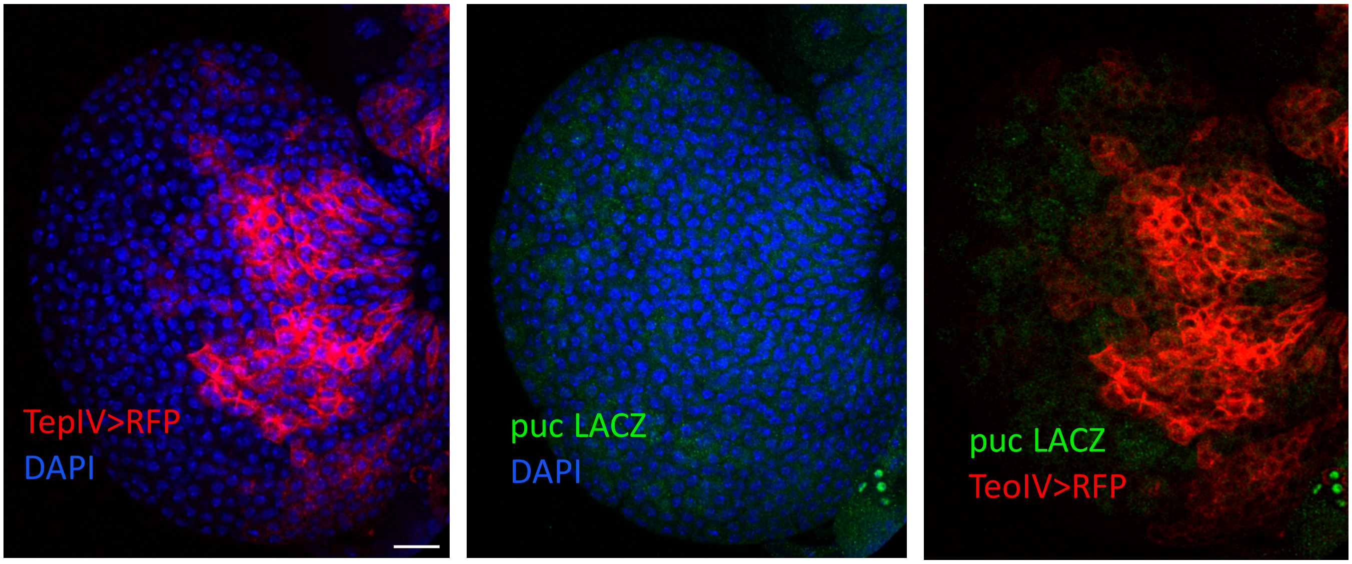

tepIV>RFP; Reli/pucLACZ

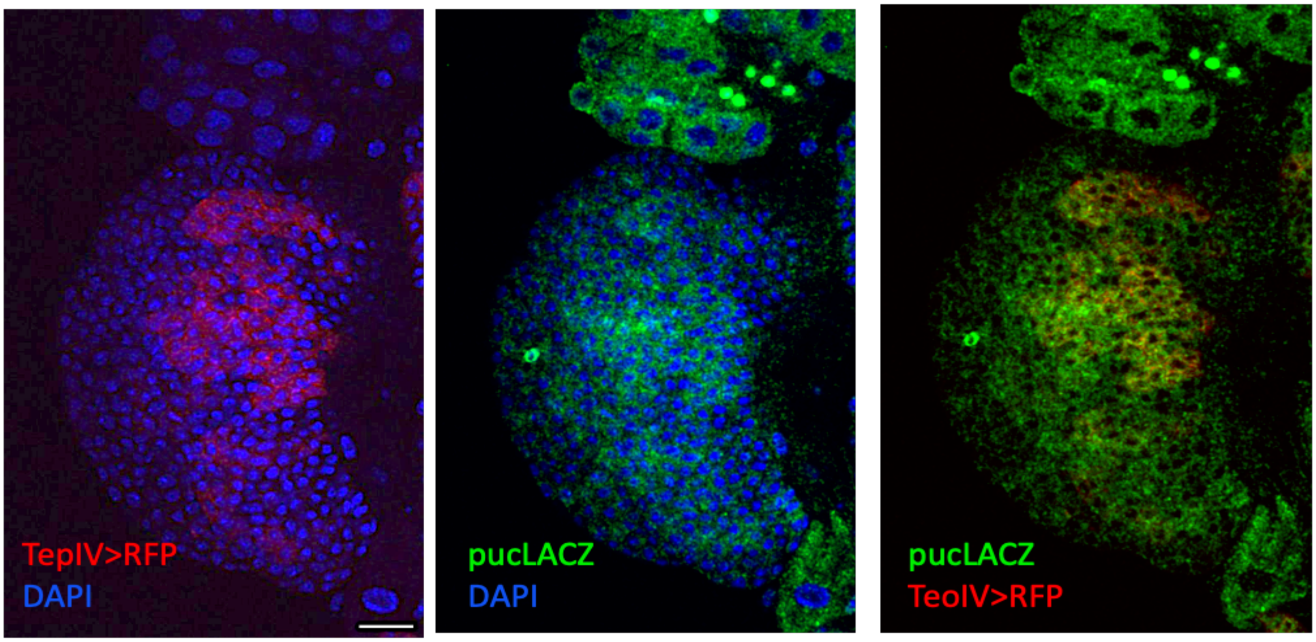

The figure legend lists the genotypes, but since no progenitor GFP expression construct is included, there is yet more confusion in this reader.

We had mentioned the construct: *tepIV-GAL4>2XEGFP* in the material and method section under Single-cell sorting and RNA isolation. Please refer to Line 610. In the revised version we have now mentioned it in the Figure legend of Figure 3A-B (lines: 834-835) and Fig 4 A-B (lines:863-864) and Figure S4 A-D (lines:1023-1024) and text Line 286.

The evidence that the mechanism through which loss of Relish promotes differentiation is via FAO-dependent acetyl-CoA-histone acetylation includes reference to their prior work (Tiwari '20) which showed that lymph gland loss of histone acetylases and/or acetyl-coA synthases blocked differentiation; that histone acetylation was reduced when FAO was blocked (including clonal analysis; and that dietary acetate supplementation (50 mM) restored histone acetylation and differentiation.

In the current paper, the authors take a more casual approach to showing that Relish loss promotes differentiation via histone acetylation. Instead of clonal analysis examining the effect of Relish expression levels on histone acetylation, the authors look at whole tissue RNAi.

We have taken the Reviewer's comments very seriously and have done clonal analyses to determine the acetylation levels in loss and gain of function of Rel. Please refer to Figure 6A-D of the revised manuscript, which shows how acetylation levels are altered due to Rel loss or OE in clonal analysis. This set of experiments further validates that Rel negatively impinges on the circuit (JNK-FAO-Acetylation), which brings about differentiation. This is also discussed in text Lines: 389-396 of the revised manuscript. We thank the Reviewer for motivating us to do the clonal analysis.

More problematic is the use of dietary acetate supplementation. Jugder et al (2021 *Immunity*) showed that dietary supplementation with the same 50 mM acetate induced Imd (Relish!) signalling in the intestine, and affected whole-organism metabolic physiology. Therefore I recommend great caution in interpreting lymph gland phenotypes resulting from acetate supplementation, because we know how much lymph gland biology is affected by immune-metabolic events elsewhere in the organism.

Our current study and the previous paper (Ramesh et al., 2021) clearly establish that the Rel expression in the lymph gland is not altered in axenic conditions. Thus, we can rule out the involvement of microbe-derived acetate in regulating Rel/IMD expression in the lymph gland.

Although lymph gland biology is dependent on the physiology of the animal in certain cases, here is one instance where the activation of the IMD pathway in the subset of the intestinal enteroendocrine cells by Microbe-derived acetate does not affect the lymph gland biology. We would also like to draw your attention to the fact that lymph glands from larvae reared in acetate-supplemented food and those reared in normal food are comparable Curent MS and Tiwari et al, 2020).

Further, the above study centers on IMD pathway signaling in EECs of the Anterior Mid-Gut that express the EEP Tachykinin (Tk+ EECs). Their Mechanistic study revealed that acetate is imported into Tk+ EECs, converted into acetyl-CoA, and used to activate the expression of ecdysone-regulated genes.

Since the expression of Rel is also independent of Ecdysone signalling in our case, we further conclude that the activation of IMD in the EECs of AMG does not affect Lymph gland biology.

Drosophila possesses 3 NF-kB factors; in addition to Relish are Dif and Dorsal. Louradour et al 2017 eLife showed that Dif expression in the lymph gland niche is activated by ROS, and contributes indirectly to progenitor differentiation into lamellocytes. Moreover, Louradour et al indicate that EGFR signalling in the progenitors synergizes with the ROS signalling to promote differentiation, but do not clarify whether this works through JNK. The authors of the manuscript under consideration do not cite this paper.

1. **This is indeed a milestone paper.**

   Our manuscript does not cite this because it elucidates Nfκ-B involvement in response to parasitism rather than a developmental context. They have shown that only upon parasitism EGFR expression is upregulated in the MZ, leading to progenitor differentiation. Interestingly, wasp parasitism increases ROS in PSC cells that activate Toll and Spitz secretion (sSpi). sSpi non-cell-autonomously activates the EGFR pathway in lymph gland progenitors (Figure 8 of Louradour et al. 2017).

   While they elucidate emergency hematopoiesis, we discuss a developmental regulation essential for progenitor homeostasis. We have now cited this in discussion. Please refer to lines 481-485 of the revised manuscript.

2. There now have been enough papers on NFkB factors in the lymph gland that each contribute some insight that there is a now need for a broader understanding of how ROS, NFkB factors, JNK interact in the lymph gland to regulate progenitor differentiation, not only into plasmatocytes, but also lamellocytes and crystal **cells, both under steady-state and immune challenge circumstances.**

   Ramesh et al. 2021, and Louradour et al. 2017, talk about the role of NFk-B factors in the lymph glands during development and infection, focussing on PSC/niche. Additionally, Khandilikar et al., 2017, have shown how "Activation of toll and imd- mediated innate immune signalling induces breakdown of the permeability barrier at the PSC", thereby affecting progenitor maintenance. The authors state, "Our work here provides a novel mechanism for the induction of the cellular arm of the immune response upon infection." Zhou et al., 2022, demonstrated that miR-317 could be regulated by Relish to fine-tune PGRP-LC expression and AMPs. This regulation leads to suppressing immune over-activation and restoring immune homeostasis.

   Since our study focuses entirely on MZ and elucidates a developmental regulation, we have not cited them. Bringing about too many signalling pathways not directly related to the phenomena we are trying to elucidate was the reason for excluding them. We have included a few of the references without disturbing the central focus of the paper, Lines 481-485.

Reviewer #3:

The Authors of this manuscript use a complex analysis by combining a genetic approach, as genetic manipulation, marker expression, morphological, gene expressional and pharmacological studies to reveal the factors and their interactions in the regulation of Drosophila blood progenitor cells. They show that Relish, an NF-ϰB-like factor, a key regulator of antimicrobial defense is a major factor too for preventing the entire progenitor pool from differentiation at one go, acting in a histologically defined region of the central hematopoietic organ. The complex analysis reveals the so far ill-defined role of Relish in regulation of hematopoiesis in normal development.

Although the multifaceted study is noticeably clearly presented and the phenotypic analysis is supported by schemes in several figures, which helps to read, a graphical abstract-like figure showing the integration of the Rel-related activation would be essential in order to help the reader to easily understand and comprehend the integration of this novel phenomenon into the already described regulatory networks.

Thank you very much for your time and encouraging comments.

We are very encouraged and satisfied that the esteemed reviewer appreciated our work.

As per your advice, we have worked on the scheme that illustrates the integration of Rel-Tak1 with already described regulatory networks to convey our findings to the readers with clarity. Please refer to Figure 7F of the revised manuscript.

We thank the three Reviewers and the editor for their time and input that has indeed enriched the paper to a large extent.
