# Supplementary material for: The NF-κB Factor Relish maintains blood progenitor homeostasis in the developing Drosophila lymph gland
Source: PLoS Genet. 2024 Sep 9;20(9):e1011403. doi: 10.1371/journal.pgen.1011403 (PMC11424005; doi:10.1371/journal.pgen.1011403)
Supplement: Attachment — Submitted filename: Response to the Reviewers comments.pdf [file pgen.1011403.s010.pdf]

Response to the Reviewers Comments:

The NF-κB Factor Relish maintains blood progenitor homeostasis in the developing *Drosophila* lymph gland. Manuscript No. PGENETICS-D-24-00160R1

Reviewer #1: The authors have adequately addressed all my concerns. It is ready for publication in PLOS Genetics.

We sincerely appreciate your time and input which have enriched manuscript.

Reviewer #2: This reviewer appreciates the thought and diligence the authors have put into this revision, particularly the clonal analysis, and the new model diagram.

We thank you for your time and suggestions which helped in enriching our work. We are glad to find that the you are satisfied with the effort put in in the revised manuscript.

In my opinion, however, the significance of this contribution would be greatly enhanced EITHER

(1) by some understanding of HOW the Relish restricted expression domain is generated, including whether other IMD pathway elements are involved,

Very interesting point raised by the esteemed reviewer.

The IMD pathway is activated when the Peptidoglycan (PGN) from gram-negative bacteria binds to the membrane-bound receptor PGRP-LC. However, recent studies have shown how IMD pathway component Relish can also be activated through developmental cues instead of the conventional pathway. In our case, neither systemic infection nor the commensal microbiome, the two major sources of circulating PGNs, has minimal effect on Relish expression in the progenitor cells. Even though there was an increase in differentiation post systemic bacterial challenge, a significant population of progenitors remained undifferentiated, which also had high Rel expression.

Interestingly, the presence of Rel in the lymph can be mapped to the embryonic stages (Berkeley Drosophila Genome Project). It is intriguing how its expression sets in as early as embryogenesis and what restricts it to the larval blood progenitor pool. Please refer to Page No: 16 and Line Nos: 474-476 where we have included this in the discussion section.

OR

(2) by a fuller discussion of the unusual nature of Relish's contribution to restraining hematopoiesis -- meaning that such an activity seems "anti-inflammatory" (tho some would disagree with this interpretation), which is opposite to the typical role that Relish and other NF-kB play. Is it similar to NF-kB role in promoting stemness in other tissues/organs/systems? In the various functions of Relish in Drosophila biology, is there any correlation between involvement of Imd pathway and type of functioning? In some ways, the lymph gland is a great tissue to understand the inflammatory vs non/anti-inflammatory functions of Relish, because it is an organ that does have such an important inflammatory function.

In our revised manuscript, we have incorporated the suggestions of the reviewers in the Introduction section Page:4, 5 Lines: .152-158. The paragraph is mentioned below:

"Although the Imd/IMD pathway has been studied intensively in immunity and inflammation, its role during fly development has only recently been unraveled. Studies have elucidated the non-inflammatory roles of Relish (Rel) in *Drosophila* neurodegeneration [14-16], apoptosis [17] and autophagy in salivary gland cells [18]. Interestingly, in the absence of infection, persistent activation of IMD in *Drosophila* gut progenitors increases the frequency of division in Intestinal Stem Cells [19]. Intrinsic NFkB activity is shown to be essential in stem cells for repairing damaged gut epithelia in *Drosophila* adult [20].

During development, Rel in the hematopoietic niche of the lymph gland is crucial for its functionality. Absence of Relish from the niche induces JNK-dependent cytoskeletal rearrangement. The aberrant cytoskeletal rearrangement perturbs the proper delivery of Hedgehog from the niche to the adjoining progenitors, leading to their precocious differentiation. Strikingly, Relish is downregulated in the niche during bacterial infection to facilitate an early dispersion of lymph gland resident hemocytes into circulation. The dynamic expression of Relish endorsed that the developmental pathway gets recalibrated in the hematopoietic niche to combat high bacterial infection [21]".

Also please refer to Lines 500-501, Page 17 where we have mentioned about the role of NFκB in HSC biology.

Is Relish's role dependent on its transcriptional activity or nuclear translocation?

The Imd pathway, homologous to the mammalian tumor necrosis factor-α (TNFα) pathway, recognizes gram-negative bacteria through membrane-bound PGRP proteins. Post-recognition follows a series of signaling events resulting in cleavage, phosphorylation, and nuclear translocation of the NF-κB protein Relish. Relish contains an N-terminal NF-κB motif and a C-terminal IκB motif. Post proteolytic cleavage, 68kD N-terminal fragment of Rel protein containing the DNA-binding Rel homology domain translocate to the nucleus and turns on the transcription of Rel-dependent genes. In our case, sustained expression of the N- terminal Relish *(UAS-Rel 68 KD)* which is known to translocate into the nucleus resulted in a halt in the differentiation program.

Our loss of function, gain of function and epistatic analyses indicate that Relish role in the blood progenitors is dependent on translocation activity. We have mentioned this in Lines:450-453, Page:15.

We thank the Editors and the Reviewers for their time and input which has greatly enhanced the quality of our manuscripts.
